# Supplementary material for: Protected 911: Development, Implementation, and Evaluation of a Prehospital COVID-19 High-Risk Response Team
Source: Int J Environ Res Public Health. 2022 Mar 4;19(5):3004. doi: 10.3390/ijerph19053004 (PMC8910754; doi:10.3390/ijerph19053004)
Supplement: Supplementary file 1 [file ijerph-19-03004-s001.zip › ijerph-1576089sup.pdf]

Table S1: Key performance indicators stratified by program week. SD = Standard Deviation. Arrival time refers to the mean interval between the arrive-scene times of the first-arriving ambulance and the HRRT crew where the HRRT crew did not arrive first or simultaneously with the first-arriving ambulance.

**Program Week**	**All Calls in System**	**Calls Involving High-Risk AGMPs**			**Capture Rate**	**Response Time** (Mean [SD])	**Arrival Time** (Mean [SD])
		Total	HRRT Attended	Missed Cases			
1	1645	31	23	8	74%	7.97 (4.15)	6.7 (8.49)
2	1457	26	18	8	69%	7.07 (3.40)	3.8 (4.79)
3	1377	28	19	9	67%	6.6 (2.47)	3.62 (7.44)
4	1406	33	27	6	81%	8.02 (4.50)	6.46 (6.84)
5	1518	25	15	10	60%	8.31 (2.31)	6.16 (7.39)
6	1560	27	21	6	77%	8.17 (3.14)	5.47 (5.81)
7	1551	22	13	9	59%	7.60 (3.34)	4.73 (5.87)
8	1491	22	19	3	86%	7.14 (3.84)	4.86 (6.46)
9	1596	16	12	4	75%	8.21 (4.89)	4.86 (6.16)
10	1695	19	12	7	63%	7.77 (3.61)	3.97 (4.71)
11	1608	18	7	11	38%	9.52 (4.38)	5.76 (5.88)
12	1239	23	12	11	52%	8.75 (3.43)	4.85 (4.33)
Total	18,143	290	198	92	67%	7.83 (4.01)	5.22 (6.56)

Table S2. Procedural success rates for advanced airway maneuvers stratified by program week. Note Advanced Airway Attempted refers to the proportion of patients in whom either endotracheal intubation or supraglottic airway insertion was attempted when indicated per the relevant medical directive(s). A successful attempt is defined as correct placement of the supraglottic airway or endotracheal tube as confirmed by (1) continuous waveform capnography; and (2) documentation of bilateral breath sounds.

**Program Week**	**Advanced Airway Attempted**	**Advanced Airway Success Rate**	**Intubation Attempted**	**Intubation Success Rate**
Pre-1	66%	85%	81%	75%
Pre-2	70%	71%	40%	0%
Pre-3	350%	85%	66%	0.0%
Pre-4	45%	88%	62%	62%
Pre-5	43%	100%	85%	100%
Pre-6	53%	100%	50%	80%
1	94%	100%	88%	88%
2	100%	94%	87%	82%
3	93%	100%	71%	76%
4	100%	100%	77%	80%
5	88%	100%	75%	85%
6	100%	92%	85%	78%
7	100%	100%	89%	100%
8	93%	100%	80%	80%
9	88%	87%	71%	71%
10	76%	100%	50%	62%
11	83%	100%	100%	100%
12	80%	100%	75%	75%
