# Supplementary figures and images for: Protected 911: Development, Implementation, and Evaluation of a Prehospital COVID-19 High-Risk Response Team
Source: Int J Environ Res Public Health. 2022 Mar 4;19(5):3004. doi: 10.3390/ijerph19053004 (PMC8910754; doi:10.3390/ijerph19053004)

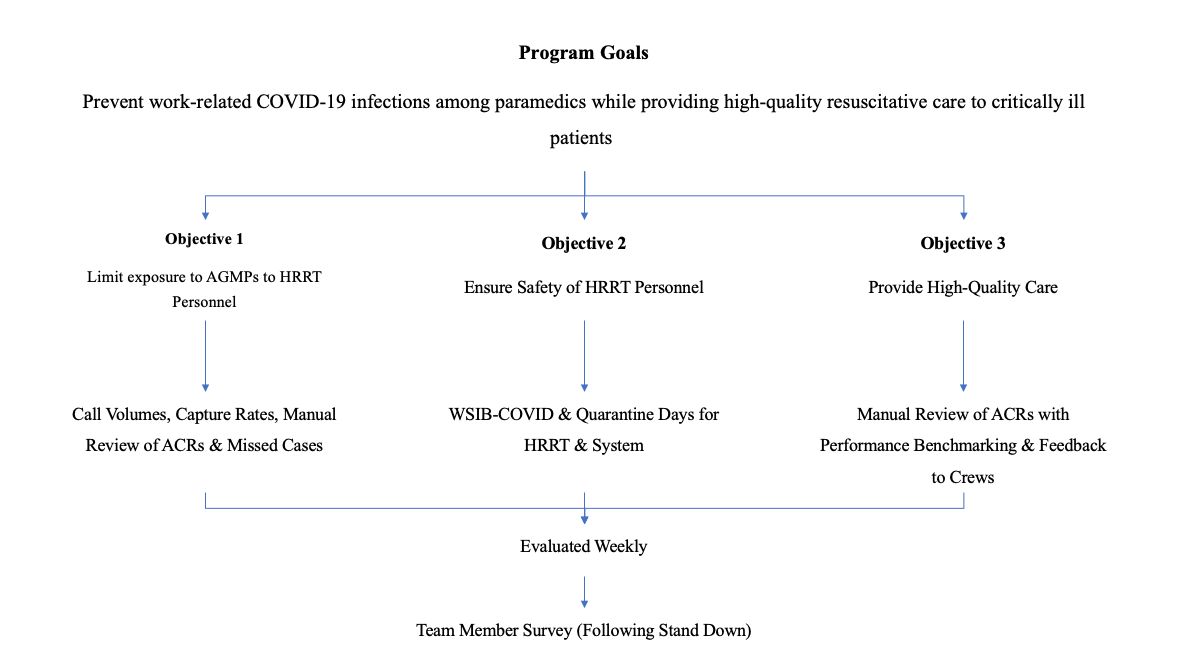

Supplement: Supplementary file 1 [file ijerph-19-03004-s001.zip › figureS1.png]
